# Putting Attention on the Spot in Coaching: Shifting to an External Focus of Attention With Imagery Techniques to Improve Basketball Free-Throw Shooting Performance

**DOI:** 10.3389/fpsyg.2021.645676

**Published:** 2021-04-16

**Authors:** Kyle R. Milley, Gene P. Ouellette

**Affiliations:** ^1^Psychology Department, Mount Allison University, Sackville, NB, Canada; ^2^Department of Athletics, Mount Allison University, Sackville, NB, Canada

**Keywords:** sport performance, motor performance, attentional focus, external focus of attention, internal focus of attention, imagery, coaching, free throw shooting

## Abstract

Attentional focus is an area that has garnered considerable attention in the sport psychology and motor performance literature. This is unsurprising given that attentional focus has been directly linked to performance outcomes and is susceptible to coaching input. While research has amassed supporting benefits of an external focus of attention (EFA) on motor performance using verbal instruction, other studies have challenged the notion that an EFA is more beneficial than an internal focus of attention (IFA) for sport-related performance. Further, it is unclear what type of instructions may serve to direct an athlete to an EFA and, in particular, if coaching can utilize imagery to orient an athlete toward an EFA. In the present exploratory study, we evaluate the effectiveness of instruction to improve free-throw shooting performance with an emphasis on an EFA brought about by implementing techniques borrowed from the imagery literature. This was tested relative to an alternate approach with an IFA induced through an emphasis on technique, devised to more closely resemble input typical of coach-to-athlete instruction. Twenty-five male and female university basketball players completed both conditions in a fully counterbalanced within-subject design. Results confirmed that participants in the EFA imagery condition had greater shooting accuracy than in the IFA technique condition. The study provides initial evidence that EFA coaching can borrow from imagery techniques, though future research should elucidate the underlying mechanisms of the effect.

## Introduction

Attentional focus is a vital component of sports given the multitude of stimuli to which an athlete must attend (Memmert et al., 2009). Where and when an athlete focuses their attention can have immense impact on their performance (Hill and Shaw, 2013). Importantly, attentional focus can be directed internally (to one's own thoughts, emotions, or physical sensations) or externally (to the environment). Within the motor learning and performance literature, an external focus of attention (EFA) refers to an individual's focus on the effects/outcomes of body movements in a motor action, while an internal focus of attention (IFA) is one's focus on the body movements themselves (Wulf, 2013). In the present study, we evaluate the immediate effects of coaching input that orients the athlete to an EFA; this is done through a novel extension of techniques borrowed from the imagery literature. This approach is tested here relative to technique-focused coaching typical of much coach–athlete interaction during training (Porter et al., 2010) that has, by virtue of its content, more of an IFA.

There is now a considerable body of research examining attentional focus in motor performance; much research has shown that an EFA leads to better motor learning and performance than does an IFA (see reviews: Peh et al., 2011; Wulf, 2013). An EFA has been demonstrated to benefit movement efficiency and effectiveness more than an IFA across several sport-related tasks. Specifically, motor learning and performance benefits have been demonstrated in balance, accuracy, maximum force production, speed, and endurance tasks, involving a wide array of sports such as golf, volleyball, soccer, basketball, swimming, and rowing (Wulf, 2013). This finding has been replicated with isolated tasks as well as with more extensive coordination of muscle groups (Peh et al., 2011). Recent research has also shown beneficial effects of an EFA for both novice and high-performance athletes (e.g., Asadi et al., 2019).

The constrained-action hypothesis provides one explanation for possible performance differences associated with EFA vs. IFA. In this explanation, an IFA may trigger conscious control which could interfere with the more efficient, automatic processing that occurs with an EFA (Wulf et al., 2001). Indeed, an EFA has been shown to reduce attentional load relative to an IFA (Kal et al., 2013). Furthermore, an internal focus may invoke the neural representation of the self, leading to self-evaluative processes that may further interfere with automatic processing (Wulf, 2013). In previous literature, this has been referred to as self-focused attention and has been linked to explicit technical coaching/instructions typical of much coach–athlete interaction (Baumeister and Showers, 1986; Liao and Masters, 2002).

Self-focused attention can also be regarded as counter to central tenets of mindfulness and flow (Csikszentmihalyi, 1990; Gardner and Moore, 2004). Flow has been described as a present moment focus similar to mindfulness, which involves effortless attention, despite immense concentration, and absorption in the task (Bernier et al., 2009; Csikszentmihalyi and Nakamura, 2010). Both mindfulness and flow states emphasize minimal internal cognitive and linguistic processing and self-evaluation (Bernier et al., 2009). Inherent to original depictions, flow state involves a quieting of the mind and a decrease in conscious self-awareness and self-focus (Csikszentmihalyi, 1990). Self-focus has been linked to sudden performance detriments (i.e., choking; Yu, 2015), and contrarily, mindfulness has been associated with the decreased susceptibility of these occurrences (Hussey et al., 2020).

There has been interest in identifying and facilitating conditions that promote mindfulness, flow, and an EFA. Jackson (1995) found that certain factors seemed to influence flow such as focus, confidence, preparation, how the performance felt and progressed, and optimal motivation and arousal. Harris et al. (2018) reported that an EFA increased perception of flow in a simulated driving task. Relatedly, Wulf and Lewthwaite (2016) presented their OPTIMAL theory to account for motor learning, which highlights the importance of an athlete's mindset, which in turn is related to attentional focus, as well as other factors including intrinsic motivation and cognitive and emotional states.

Despite the theoretical and empirical support favoring an EFA over an IFA for sport performance outcomes, there remains some debate as to the strength of this evidence. At the forefront of this debate, Toner and Moran (2015, 2016) maintain that an IFA is needed to make high-level athletes aware of the kinesthetic discrepancies between desired and actual movement; yet, it has been suggested that an EFA does not mean that athletes are completely unaware of their movements, but rather that the primary focus is on the effects of that movement while preparing for movement execution (Wulf, 2015). Still, it must be noted that some recent studies have found only limited impact of EFA on measured performance (e.g., Harris et al., 2018). Further, much of the research in this area has suboptimal ecological validity as it is often laboratory-based, with great variation in length of interventions and how the outcomes are measured (for examples, see Wulf, 2013; Wulf and Lewthwaite, 2016).

Ecological validity in sport research can be maximized by studying attentional focus within natural sporting contexts and environments, while directly evaluating the immediate effects of coaching input on athlete performance. This is especially important considering that a coach may unknowingly induce an IFA over an EFA by overemphasizing technical instructions. Despite evidence of the benefits of an EFA, mindfulness, and flow, increased pressure to win in more competitive sport may lead to more controlling, prescriptive coaching, often high in IFA (Porter et al., 2010). In many ways, this represents a coaching paradox in terms of practice not aligning with research. One potential means of shifting coach–athlete interactions toward more of an EFA can be achieved through techniques borrowed from imagery interventions within the psychological skills training literature.

Imagery is the mental simulation of real experience involving the combination of different sensory modalities (kinesthetic, visual, auditory, olfactory), which allows one to represent perceptual processes in one's mind without the actual sensory stimuli, input or motor movements (Munzert et al., 2009; Fazel et al., 2018). In the context of sport, imagery can complement coaching to the benefit of a multitude of different outcomes, including motor learning and performance, tactical movements, motivation, self-confidence, anxiety, strategies, problem solving, and rehabilitation. Athletes may use imagery prior to, during, or after practices and competitions, as well as during rehabilitation (Guillot and Collet, 2008; Cumming and Williams, 2013).

The Revised Applied Model of Deliberate Imagery Use (RAMI; Cumming and Williams, 2013) outlines many areas that may benefit from imagery while specifying key components of imagery methodology. The RAMI identifies recommended aspects of imagery use, including the “who,” “where,” “when,” “why,” and “how.” A further personalized approach to imagery acknowledged by the RAMI is the PETTLEP model (Holmes and Collins, 2001). PETTLEP stands for physical, environment, task, timing, learning, emotion, and perspective. Each facet of the model is based on the idea of functional equivalence—or that the motor imagery system is fundamentally related to the motor preparation and execution system of the brain. The imagery environment, internal (first-person) or external (3rd person) visual perspective, and timing should be as close as possible to the one where the actual motor action occurs. Indeed, research has shown that imagery activates similar brain regions as movement execution (Mizuguchi et al., 2012) and has been used to prime the desired approach of a subsequent action (Stoykov and Madhavan, 2015).

Within the domain of psychological skills training, imagery approaches typically do not explicitly consider the role of attentional focus (e.g., see Holmes and Collins, 2001; Cumming and Williams, 2013). As a result, scripts implemented in some research (e.g., Fazel et al., 2018) may unintentionally trigger an IFA due to a focus on body movements. The disconnect between imagery and attentional focus literature may be attributed to imagery being regarded as a longer-term intervention in the domain of psychological skills training, separate from coaching instruction in the domain of attentional focus and athlete performance. Indeed, in a meta-analysis by Cooley et al. (2013), studies that were <5 days duration were not included, as they were perceived to not meet the duration criteria for an imagery intervention.

Yet, there are techniques and aspects of imagery that are amendable to short-term, even on-the-spot, Coach–athlete interactions (Leong et al., 2019). In particular, the RAMI and PETTLEP models suggest methodology that can be implemented specifically to shift an athlete to an EFA through more effective and efficient priming of task-relevant stimuli. Of interest here is how that would impact immediate athlete performance relative to traditional coaching of technique that by nature is oriented more to an IFA. We know of only one such study that has directly compared the implementation of imagery techniques specifically devised to have a high EFA with more typical technical coaching that would have a high IFA, albeit spread out over a prolonged period of time (16 weeks). Guillot et al. (2013) reported that an instructional condition of imagery techniques with a deliberate EFA improved performance on tennis serve performance relative to more traditional technique coaching. It should be noted, however, that this was a small-sample study (*N* = 12), with youth participants only (aged 11 years), and critically the imagery condition was always delivered last in a within-participant design (i.e., there was no counterbalancing). Hence, more research in this understudied area is clearly needed.

## The Present Study

We had collegiate-level basketball players complete a free-throw shooting task with instruction designed to promote an EFA through implementation of techniques borrowed from imagery interventions. We opted for a short-term, in-season, single session that took place in the team's regular practice facilities to increase ecological validity; many real-life sport scenarios may benefit from imagery techniques to bring about an EFA but are limited in time (a single practice, before a game, half time, etc.). These same athletes completed a comparison condition (within a fully counterbalanced experimental design), devised to reflect more prescriptive coaching with a high IFA (by emphasizing technique), as often associated with traditional coach–athlete interactions during training. Due to the potential combined benefit of task-relevant multimodal priming (Stoykov and Madhavan, 2015) and optimized attentional focus (Wulf, 2013), it was hypothesized that participants would have better free-throw shooting performance in the EFA imagery condition than in the IFA technique condition. Given the potential overlap between flow and attentional focus as suggested by current theory (e.g., Wulf and Lewthwaite, 2016), flow state measures were also completed after each condition to evaluate any influence on the athletes' perception of flow.

## Materials and Methods

### Participants

Twenty-six Canadian Collegiate Athletic Association basketball players originally participated in the study. During the EFA imagery session for one participant, there was considerable disruptive noise in the gymnasium and the participant reported having difficulty focusing; they were excluded from the data analysis reported here. Remaining participants were 9 male and 16 female university students aged 18–24 years (*M* = 19.92, *SD* = 1.48). Participants reported a mode of 10+ years' basketball experience. All participants had at least moderate imagery ability as determined by a screening measure (MIQ: Movement Imagery Questionnaire-3; Williams and Cumming, 2011). Institutional ethical approval was granted, and all participants provided informed consent. These players were members of a university varsity basketball program; all members of both the men's and women's teams were asked to participate; the sample reported here were those who consented.

### Materials

#### Flow State Scale-2

Flow is described as an optimal state of consciousness involving a challenge-skills balance; action-awareness merging; clear goals; unambiguous feedback; concentration on task; sense of control; loss of self-consciousness; time transformation; and autotelic experience (Csikszentmihalyi, 1990). The short version of the Flow State Scale-2 (FSS-2) was used to measure state flow following each condition as it is intended for use after an event to assess state flow experience (Jackson, 2002). FSS-2 has acceptable validity and reliability (*r* = 0.76–0.90; Jackson et al., 2008).

#### Free-Throw Performance

In basketball, a fouled player shoots one to three free throws from the free-throw line. Players typically score one point if the basketball goes through the basket. The free-throw line is 4.22 m away from the 0.45-diameter circular basketball rim, which is 3.05 m above the floor. The current study replicated the scoring system by VaezMousavi and Rostami (2009) to measure free-throw accuracy more precisely: 3 points for a basket entering the hoop without touching the rim or backboard, 2 points for a basket that touches the rim or backboard before going in, 1 point for hitting the rim or backboard without scoring a basket, and 0 points for not scoring a basket, touching the rim or the backboard. Each shot was scored live by a researcher.

#### EFA Imagery Condition

Instruction within this condition was created with reference to the PETTLEP Model (Holmes and Collins, 2001; Wakefield and Smith, 2012) and the RAMI (Cumming and Williams, 2013). Participants received instruction in the gymnasium wearing their practice clothes, holding a basketball in their hands; participants were instructed to image in real time. These instructions were consistent with the Physical, Environment, and Timing elements of the PETTLEP model while increasing ecological validity. Consistent with the PETTLEP and the RAMI, participants were instructed to try to feel physical movements as they occur and see, hear, and feel as they would in the real world. These instructions were designed to enhance the realism of the task and improve functional equivalence. Participants were instructed to “image through your own eyes” according to information regarding the perspective element of PETTLEP (Wakefield and Smith, 2012). After hearing the instructions, participants listened to a recorded imagery script (recorded to add consistency across participants), using a Tascam Dr-40 player with professional quality (PSB) noise-canceling headphones. A female voice was used to record the script.

The script itself consisted of two halves, each with two sections (for a total of four blocks), and was designed similarly to the retrogressive imagery script in Fazel et al. (2018) in that it transitioned from extensive contextual information in the first half to minimal contextual information by the last, to enhance the selective attention on task-relevant stimuli. This also corresponded with the learning element of PETTLEP, as each section was altered to enhance focus on the outcome of shooting free throws. Scripts included direct instructions such as “…you take a deep breath and begin your free throw routine. As you do your routine, your attention remains focused on the net. As you release your shot, you visualize it going in the basket. You watch the ball soar through the air and drop perfectly through the netting. The sound indicates it was a swish…” Full transcripts of the imagery are available from the authors.

#### Imagery Manipulation Check

Participants were asked to indicate how well they saw, heard, felt, and experienced their imagery on a zero (not at all) to four (very well) Likert Scale.

#### IFA Technique Condition

Similar to the self-focused attention design used by Liao and Masters (2002), in this condition participants were instructed to “be aware of what you are doing” and “pay close attention to the mechanics of your shooting process” in order to induce an IFA before shooting free throws. They were told to approach the free throws as they would during an intense basketball game to give context and adding ecological validity to the task. They were provided with a list of technical instructions used by Zachry et al. (2005), intended to reflect technical tips or feedback that would be typical in direct coaching, and were told to review aspects of their free-throw technique before each shot. There were nine related technical aspects provided on a sheet that involved reference to stance, grip, or mechanics of shooting a free throw. They were not directly told to focus on all the techniques provided in the list; instead, the techniques were there to encourage them to remember technical aspects of free-throw shooting as relevant to their own performance (i.e., intended to bring more focus to their own body mechanics).

#### IFA Manipulation Check

The IFA manipulation check served two purposes: To be a statistical manipulation measure and to further induce an IFA for remaining trials. Participants were asked to indicate how often they focused on their technique when shooting free throws on a zero (never) to four (all the time) Likert Scale. Then, they were instructed to “recall their technique” and were given a space to write.

### Procedure

All participants were asked to fill out a demographic information form and the Movement Imagery Questionnaire at time of consent to ensure participants had at least moderate imagery ability for participation in the study. Then, they were randomly scheduled into one of two gender-stratified conditions and counterbalanced in a within-subject design. The conditions were the EFA imagery condition and the IFA technique condition. Participants completed the subsequent condition after a minimum of 1 week and a maximum of 2 weeks. All sessions were conducted individually and in the same university gymnasium where the players played their home league.

Each condition involved a single training session of free-throw shooting, consisting of 10 baseline shots followed by four performance blocks, each consisting of 10 shots. To minimize the number of necessary statistical tests and to correspond to the two halves of each intervention, shooting performance was summed across the first two blocks for a score for the first half of the training session and summed across blocks 3 and 4 to yield a score of shooting accuracy for the second half of each session.

In the EFA imagery condition, participants were given instructions on how to image, following completion of 10 baseline shots and an explanation of the scoring method. Participants then listened to the first of four segments of the recorded imagery script, following which they completed the first of four blocks of 10 performance shots. After every 5th performance shot, participants were reminded to “focus your attention on the ball going into the basketball net.” On each consecutive segment, the script provided progressively less contextual information to focus the participant's attention on the outcome of shooting. This procedure was repeated for each half of the imagery script, with each half containing two segments/blocks of performance shots (i.e., 10 shots per block; 20 shots per half). After the last block was completed, the imagery manipulation check and the short version of the Flow State Scale-2 were given.

In the IFA technique condition, participants were similarly instructed on the scoring method and completed 10 warm-up shots. They then completed a total of four blocks of 10 shots, with reminders following every five shots, as in the imagery condition. Participants were given the IFA technique instructions including directives meant to induce an IFA during free-throw shooting such as “be aware of what you are doing” and “pay close attention to the mechanics of your shooting process.” They were also told to review a list of technical aspects of free-throw shooting before each block. After every five free throws were completed, the participant was reminded to focus on their technique. To serve as a manipulation check and further promote an internal focus of attention, participants were asked to recall as much as possible about their shooting processes after each half of the intervention. They were also asked to complete a Likert Scale indicating the degree to which they focused on technique. This process was concluded by asking participants to fill out the short version of the Flow State Scale-2 at the end of the session.

## Results

**Figure 1:**
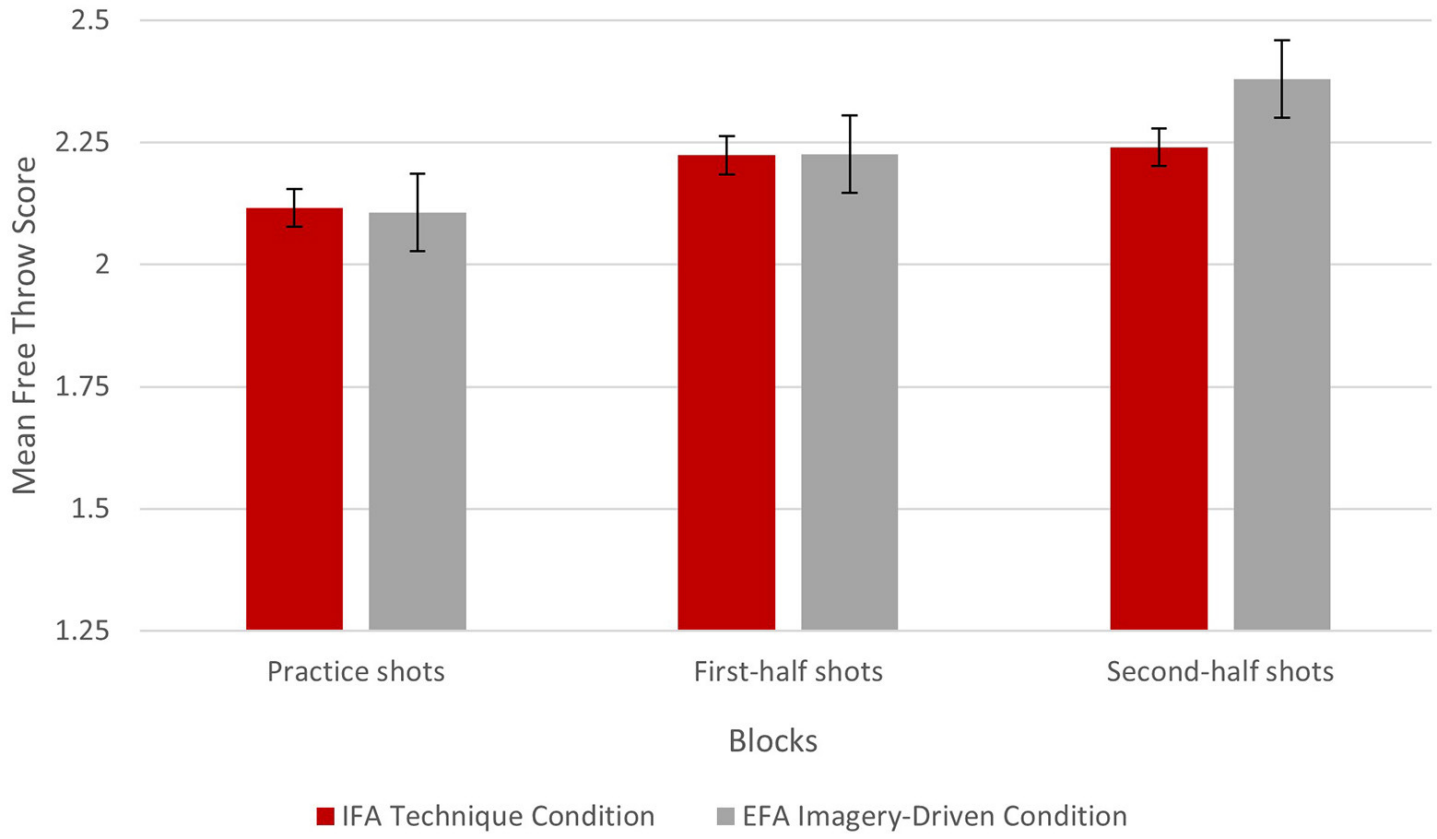
Mean free-throw scores across blocks and conditions.

Participant scores on the Movement Imagery Questionnaire (*M* = 5.78, SD = 0.68), Imagery Manipulation Check (*M* = 3.10, SD = 0.47), and IFA Manipulation Check (M = 3.23, SD = 0.47) were deemed to be acceptable for inclusion in analysis and confirmed the fidelity of the interventions. Means and standard deviations for key constructs are presented in Table 1, along with bivariate correlations to allow for an examination of relations among measures. Shooting performance across conditions is shown in Figure 1.

**Table 1:** Descriptive statistics and correlations.

**Measure**	**1**	**2**	**3**	**4**
1. Flow (IFA)	–			
2. Flow (EFA)	0.523^**^	–		
3. IFA trials	0.477^*^	0.215	–	
4. EFA trials	0.470^*^	0.352	0.773^**^	–
*M*	4.146	4.129	2.206	2.282
*SD*	0.329	0.370	0.243	0.251

*N = 25 for all constructs. The numbers associated with the variables on the first column correspond with the numbers on the top row*.

* *Indicates significance at the p < 0.05 level*.

** *Indicates significance at the p < 0.01 level*.

*Flow (IFA), Dispositional Flow State Scale-2 administered after final shots in the IFA technique condition. Flow (EFA), Flow State Scale-2 administered after final shots in the EFA imagery condition. IFA Trials, free-throw shooting scores averaged across all performance shots in the IFA technique condition. EFA Trials, free-throw shooting scores averaged across all performance shots in the EFA imagery condition*.

To address the central question of the present investigation, a 2 (EFA imagery vs. IFA technique condition) × 3 (baseline shot performance, performance over the first half of intervention, performance over the second half of intervention), repeated-measures analysis of variance (ANOVA) were conducted. The assumption of sphericity was not violated. Of worthy is that the main effect of condition was significant with a medium effect size, [*F*_(1, 24)_ = 2.96], *MSE* = 0.05, *p* = 0.048 (one-tailed for hypothesis testing), *η*^2^ = 0.111.

As indicated in Figure 1, the main effect of trial was also significant with a large effect size, [*F*_(2, 23)_ = 4.76], *MSE* = 0.06, *p* = 0.019, *η*^2^ = 0.293. The interaction between condition and trial was also significant with a large effect size, [*F*_(2, 23)_ = 3.14], *MSE* = 0.04, *p* = 0.031, *η*_*p*_^2^ = 0.214.

To further explore the significant interaction, *post-hoc* LSD tests were conducted. Importantly, it was necessary to confirm equivalent performance prior to the start of each condition: this comparison revealed no statistically significant difference between the conditions for performance on the baseline shots (*p* = 0.587). Further comparisons revealed no statistically significant differences between conditions on shooting performance in the first half of the interventions (*p* = 0.968). By the second half of the interventions, however, superior performance was evident for the EFA imagery condition with a very large effect size, [*F*_(1, 24)_ = 12.85] *MSE* = 0.02, *p* < 0.001, *η*^2^ = 0.349.

Moreover, pairwise comparisons within the IFA technique condition indicated no significant differences between any test points (*p*'s = 0.131–0.786). Pairwise comparisons within the EFA imagery condition showed a significant improvement between the performance on the baseline shots and performance at the second half of the intervention [*F*_(1, 24)_ = 12.46 *MSE* = 0.05, *p* = 0.001, *η*^2^ = 0.342], as well as between performance during the first and latter halves of the intervention [*F*_(1, 24)_ = 298 22.78 *MSE* = 0.01, *p* < 0.001, *η*^2^ = 0.487].

Turning to Table 1, there was a positive relationship between the scores from the Flow State Scale-2 administered after the final shots in the IFA technique condition and performance in both conditions, *r's* = 0.470–0.477. A similar relationship was not seen for the Flow State Scale-2 administered after the EFA imagery condition; this flow measure was not significantly correlated with shooting performance although the trend was approaching significance (*p* = 0.145). A repeated-measures analysis of variance was also conducted to examine the effect of imagery condition on Flow State Scale-2 scores. The main effect of condition was not significant, [*F*_(1, 24)_ = 0.650], *MSE* = 0.06, *p* = 0.801, indicating that athletes reported relatively high flow states after training within each condition.

## Discussion

The present study examined the effect of an athlete's focus of attention during a basketball free shooting training session in an ecologically valid implementation of a counterbalanced within-participant experimental design. The comparison of interest was the immediate effects on shooting accuracy brought about by a mode of instruction borrowed from imagery interventions with the purpose of directing the athlete to an EFA vs. a more traditional technique-oriented session that by definition would have a high IFA. Effects on immediate flow state were also evaluated within each instructional condition. Results showed that the EFA imagery condition produced better free-throw shooting than the IFA technique comparison, with the improvement in shooting accuracy becoming apparent by the latter half of the training session. These findings demonstrate that imagery techniques can be implemented within a sport practice environment and support the contention that an EFA is beneficial over an IFA for sport performance, at least in the short term. The results regarding flow, however, were more convoluted; contrary to theory that links an EFA to flow, there was no significant difference in flow state between conditions; yet it should be noted that a high flow state score was reported in both.

### Free-Throw Performance

The results of the current study align with the limited research involving the use of imagery techniques to elicit an EFA (Guillot et al., 2013). However, unlike previous research, the current study involved older, highly experienced athletes with the goal of maximizing immediate performance in a short duration. It is notable that there was a significant difference in free-throw shooting performance between conditions considering that the training conditions of the current study were each conducted over a single session with a duration not exceeding 30 minutes. In the meta-analysis of imagery interventions by Cooley et al. (2013), studies that were <5 days in duration did not meet criteria to be included in the study given the expectation that imagery interventions are more long term by nature. Yet, in competitive sport there are many situations in which techniques derived from imagery interventions may be beneficial to an athlete facing time constraints (e.g., half-time, during a substitution, immediately before a match, etc.); hence, it is especially noteworthy that the EFA condition devised for the present study induced improved performance outcomes over such a short time period. This may well speak to the power of a brief EFA imagery approach to coaching instruction to maximize the performance of experienced, competitive athletes.

### Flow

Flow experience has been linked to performance across multiple sports (Bakker et al., 2011; Koehn, 2017). The current study found only partial support for this relationship as flow state was correlated with performance in the IFA condition, but not within the EFA training condition. However, this was approaching significance within the EFA condition and the lack of statistical significance is likely an artifact of sample size and power. It is also noteworthy that no difference in flow state scores was found across conditions; flow states were high within both conditions, as evidenced by mean scores reported within the conditions. Note that the interpretation of the flow measure, as guided by the test material, is that higher values (maximum of five) indicate a strong agreement of flow experience. Hence, it may well be that while the EFA training condition succeeded in improving shooting accuracy, it did not increase the flow state of the participants relative to the IFA technique comparison condition due to the overall high flow states of these athletes in general.

The constrained-action hypothesis has been proposed to explain why an EFA may be preferable to an IFA when it comes to motor learning and performance (Wulf, 2013). In particular, it is thought that an IFA may interfere with more efficient, automatic processing by triggering self-evaluative processes (Wulf et al., 2001). Given that self-evaluative processes are counter to establishing and maintaining flow states (Harris et al., 2018), the high flow state scores within our IFA training condition may seem surprising. Indeed, Harris et al. (2018) reported that an EFA increased flow but not performance in their study involving a driving simulation, which is opposite to the pattern reported here. This discrepancy may result from the high experience and skill level of participants included in the present study, in conjunction with what was a familiar task in a low-pressure environment. Memmert et al. (2009) observed that experts were better than novices at switching between attentional modalities; their experience allowed them to pay attention to what was most important during a sport task. Therefore, the extensive basketball experience of the high-level athletes included in the current study may have made them less susceptible to self-evaluative processes during the preparation or completion of a motor action, thereby protecting against any threat to flow associated with an IFA.

The IFA technique condition employed here also likely provided participants with an optimal skill-challenge zone as they engaged in technical behaviors within their realm of expertise and ability. Indeed, expert musicians experienced flow as a function of certain self-regulated practice behaviors (Araújo and Hein, 2016). It is possible that experienced individuals can self-regulate the demands of a familiar task in a practice environment, thereby promoting flow. Interestingly, an optimal skill-challenge environment is a dimension of flow described by Jackson (1995). It is possible that the design of the study did not provoke external stressors, which would have likely been detrimental to flow (Baumeister and Showers, 1986), and instead encouraged a skill-challenge balance, which may have served to maintain the flow state of participants during the IFA condition.

### Limitations and Directions for Future Research

The present results demonstrate a significant improvement in free-throw shooting following a single, brief session that employed imagery techniques to elicit an EFA. Despite the encouraging results of our intervention, it is difficult to precisely isolate the underlying mechanisms driving the effect reported here. As previously described, while performance increased more with the EFA imagery instruction, flow did not (resulting in a lesser correlation between flow and performance within this condition). Given the performance benefits seen over the IFA technique condition, the shift to an EFA would thus seem to be implicated as the driving factor.

Yet, as acknowledged earlier we prioritized ecological validity in comparing an EFA condition that included guided imagery to an IFA condition that focused on technique, as our interest was to have a comparison condition that would resemble more typical coaching (as per Guillot et al., 2013). While this provides a valuable comparison for practical applications, it does limit our ability to isolate a single causal factor precisely; in this respect, more research in this important area is warranted. It would be informative in future research, for instance, to manipulate attentional focus within different imagery interventions; this is especially relevant given that the role of attentional focus is near-absent from both the RAMI and PETTLEP models. Thus, while recent research has supported the use of imagery within coaching (Leong et al., 2019), the current study highlights the need to better elucidate EFA and IFA coaching instruction embedded within imagery. Future research may also target differential effects on athletes of different ages and levels of ability, and compare performance within the training study paradigm itself with performance within subsequent game-level competition.

Distinguishing the underlying mechanisms is further complicated by research that has documented beneficial outcomes following motor imagery practice, which may well invoke an IFA through covert movement rehearsal (see Moran and O'Shea, 2020). However, it is important to note that the kinesthetic sensations involved in motor imagery are not necessarily inherent to an IFA. When a particular skill is well-practiced, external visual cues may prime the kinesthetic sensations associated with a task. This may well lead to more effective and efficient consolidation of kinesthetic stimuli in accordance with demands of the task, while avoiding any detriments associated with an IFA. In this way, we speculate that our findings do not directly contradict those of much motor imagery literature but instead highlight the importance of investigating how task-relevant implicit and explicit kinesthetic sensations may interact with athlete experience to influence performance.

Nevertheless, the results of our short-term intervention are suggestive in terms of coaching applications. Our results align with the body of research demonstrating the benefits of an EFA over an IFA and show how this can be brought about within a single training session by employing techniques borrowed from imagery interventions. While more work is required to clarify the theoretical basis of the current results, the practical applications are certainly intriguing.

## Data Availability Statement

The raw data supporting the conclusions of this article will be made available by the authors, without undue reservation.

## Ethics Statement

The studies involving human participants were reviewed and approved by REB Mount Allison University. The participants provided their written informed consent to participate in this study.

## Author Contributions

KM devised/compiled the materials, recruited the participants, and oversaw the interventions and data collection. Both authors were involved in the data analysis, preparation of this manuscript, conceptualization, and design of the research presented here.

## Conflict of Interest

The authors declare that the research was conducted in the absence of any commercial or financial relationships that could be construed as a potential conflict of interest.
